# Effect of Dietary Acidolysis-Oxidized Konjac Glucomannan Supplementation on Serum Immune Parameters and Intestinal Immune-Related Gene Expression of *Schizothorax prenanti*

**DOI:** 10.3390/ijms18122558

**Published:** 2017-11-28

**Authors:** Mingrui Chen, Shuyao Wang, Xue Liang, Donghui Ma, Li He, Yaowen Liu

**Affiliations:** 1 College of Food Science, Sichuan Agricultural University, Yaan 625014, China; 18283584025@163.com (M.C.); shuyaow@163.com (S.W.); xue6liang17@163.com (X.L.); 18894314357@163.com (D.M.); helifood@sicau.edu.cn (L.H.); 2 School of Materials Science and Engineering, Southwest Jiaotong University, Chengdu 610031, China

**Keywords:** *Schizothorax prenanti*, acidolysis-oxidized konjac glucomannan, immunity

## Abstract

The present study was conducted to investigate the effects of dietary acidolysis-oxidized konjac glucomannan (A-OKGM) (0%, 0.4%, 0.8%, and 1.6%) supplementation on the immunity and expression of immune-related genes in *Schizothorax prenanti*. After feeding for eight weeks, the serum and guts were used for measurement of biochemical parameters, and immune-related gene expression in the gut were also analyzed by real-time quantitative polymerase chain reaction (RT-qPCR). C-reactive protein and IgM levels were significantly higher in the A-OKGM fed groups than in the control group, regardless of the dosage. The 0.4% and 1.6% A-OKGM groups showed significant up-regulation of tumor necrosis factor α (*TNFα*) in the anterior gut. The 0.8% and 1.6% A-OKGM groups also showed significantly enhanced *TNFα* expression in the mid- and distal guts. Interleukin-1β (*IL-1β*) expression in the anterior gut of fish fed with 0.4% and 1.6% A-OKGM diets was significantly enhanced. The 0.8% and 1.6% A-OKGM diets resulted in significantly increased the expression of *IL-1β* in the distal gut. Similarly, the interleukin-6 (*IL-6*) messenger RNA (mRNA) levels in the 0.4% and 1.6% diet groups were significantly higher in the anterior gut. The 0.8% and 1.6% A-OKGM diet groups showed significant induction of *IL-6* gene expression in the distal gut. A-OKGM modified from KGM can act as an immunostimulant to enhance the immunity of *S. prenanti*.

## 1. Introduction

*Schizothorax prenanti*, also known as ‘ya fish’ (Cypriniformes, Cyprinidae, Schizothoracinae), is a cold-water fish endemic to the southwestern part of China. It reproduces mainly in the upper reaches of the Yangtze River and the Hanjiang River. *S. prenanti* is considered to be a good candidate for freshwater aquaculture in China due to its delicious meat and high market value. However, *S. prenanti* is susceptible to different diseases, such as infection from motile *Aeromonas septicaemia* [1], which can affect the quality of its flesh. Recently, the problem of infected fish has been reported from some fish farms. Hence, finding a solution to improve the intrinsic immunity of this fish is an urgent need.

The intestine, as a multifunctional organ, is responsible for nutrition uptake and pathogen recognition. However, when it comes to the immunostimulatory effects of prebiotics, or disease resistance studies, the intestine has received less attention than other immune organs, such as the head, the kidney, and the spleen [2]. Research on the intestinal immunity of fish is of special interest for the fish farming industry for several reasons. First, farmed fish kept at high breeding densities are susceptible to numerous intestine-related diseases, because the intestine is the main entry point for harmful microorganisms [3]. Second, the gut microbiota, which is a potential factor for modulating fish pathogens, is likely to respond to dietary manipulations [4,5]. Third, farmed fish are typically fed commercial pellet feeds, which provide opportunities for manipulating intestinal health through the incorporation of various food additives in the feed [6]. Therefore, understanding of diet intestine interactions is necessary for the development of new strategies to prevent and treat various types of infectious diseases in fish.

Konjac glucomannan (KGM), which is derived from the tuber of Amorphophallus konjac, is a type of prebiotic. To date, many authors have studied the effects of prebiotics on the immunity of fishes [7,8,9]. However, the high molecular weight (500–2000 kDa) and viscosity of KGM impede its application in the aquafeed industry. As such, we produced oxidized konjac glucomannan (OKGM), which is an oxidative degradation product of KGM, with higher purity and lower molecular weight. However, the molecular weight of OKGM was still found to be high. Therefore, hydrochloric acid (HCl) and hydroperoxide (H_2_O_2_) were used as degradation reagents to produce acidolysis-oxidized konjac glucomannan (A-OKGM), whose molecular weight is lower than that of OKGM. Previous studies have shown that the molecular weight of A-OKGM is 9.8 kDa, and the functional groups of A-OKGM are same as that of KGM [10]. 

In this study, we investigated the effects of A-OKGM on the expression of immune related genes in the anterior intestine, mid-intestine, and distal intestine of *S. prenanti*. Serum was also collected from the fishes to analyze the IgM level, C-reactive protein (CRP) level, and lysozyme activity. The results have implications for preventing infectious diseases in *S. prenanti*.

## 2. Results

### 2.1. Visceral Index

As shown in Table 1, the spleen index in fish fed with 1.6% A-OKGM was significantly different (*p* < 0.05) from that in the other groups. Similarly, the liver index in fish fed with 0.8% A-OKGM was markedly higher (*p* < 0.05) than that in the control group. The head kidney index, gut index, and mesonephros index did not vary significantly among all of the groups (*p* > 0.05). 

### 2.2. Immune Parameters

**Figure 1:**
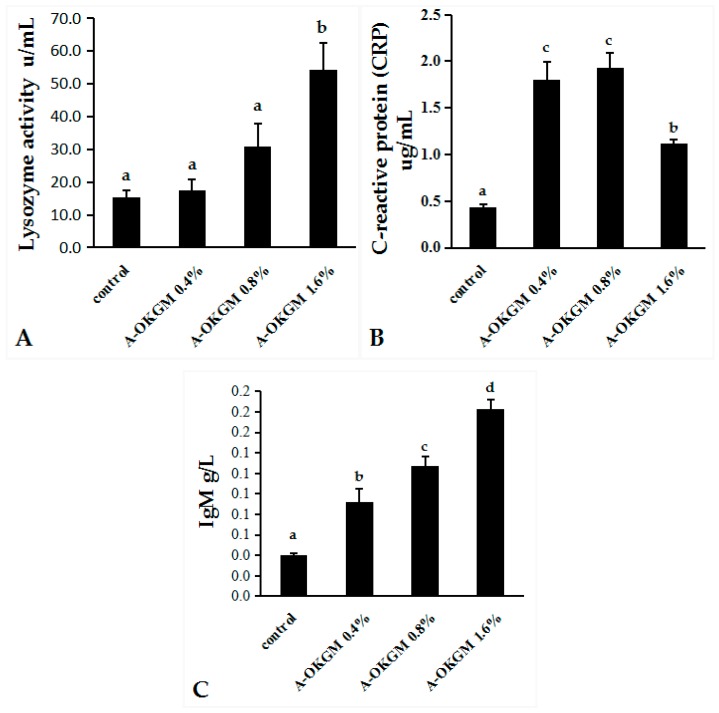
(**A**) Lysozyme activity, (**B**) C-reactive protein level and (**C**) IgM level of *Schizothorax prenanti* after consumption of the experimental diet. The observed values are expressed as the mean ± standard error (S.E). ^a–d^ Means with different superscripts are significantly (*p* < 0.05) different from each other.

As is shown in Figure 1, the CRP levels in fish fed the A-OKGM diets were significantly higher (*p* < 0.05) than those in the control group, regardless of the dosage. The IgM contents were significantly increased by the A-OKGM diets (*p* < 0.05), with the highest IgM level observed in the 0.8% A-OKGM diet group. Lysozyme activity in fish fed the 1.6% A-OKGM diet was significantly increased (*p* < 0.05). However, the lysozyme activity did not significantly vary among the other groups.

### 2.3. S. prenanti Tumor Necrosis Factor α (TNFα) mRNA Expression

**Figure 2:**
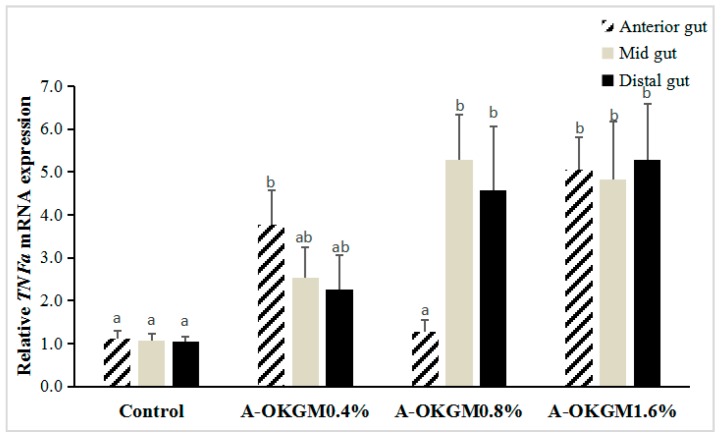
Effects of A-OKGM on *TNFα* expression in the anterior, mid- and distal gut of *Schizothorax prenanti*. The observed values are expressed as the mean ± S.E. ^a,b^ Means with different superscripts are significantly (*p* < 0.05) different from each other.

In the anterior intestine, *TNFα* expression in fish fed the 0.4% and 1.6% A-OKGM diets was significantly increased (*p* < 0.05). In the mid-intestine, the *TNFα* mRNA levels in fish fed the 0.8% and 1.6% A-OKGM diets were significantly higher (*p* < 0.05) than those in the control group. In the distal intestine, *TNFα* expression was significantly increased (*p* < 0.05) by both the 0.8% and 1.6% A-OKGM diets. These results are depicted in Figure 2. 

### 2.4. S. prenanti Interleukin (IL)-1β (IL-1β) mRNA Expression

**Figure 3:**
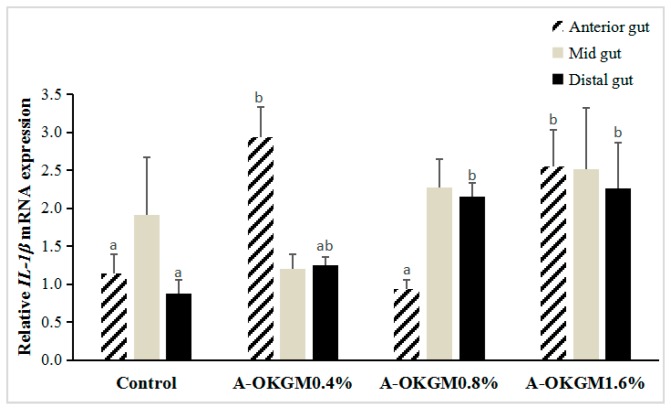
Effects of A-OKGM on *IL-1β* expression in the anterior, mid- and distal gut of *Schizothorax prenanti*. The observed values are expressed as the mean ± S.E. ^a,b^ Means with different superscripts are significantly (*p* < 0.05) different from each other.

In the anterior intestine, the expressions of *IL-1β* in fish fed with 0.4% and 1.6% A-OKGM diets was significantly increased (*p* < 0.05) when compared with that in the other groups. However, the increase in *IL-1β* expression in the mid-intestine did not reach significance (*p* > 0.05). In the distal intestine, the 0.8% and 1.6% A-OKGM diets significantly promoted (*p* < 0.05) the expression of *IL-1β*. These results are depicted in Figure 3.

### 2.5. S. prenanti Interleukin-6 (IL-6) mRNA Expression

**Figure 4:**
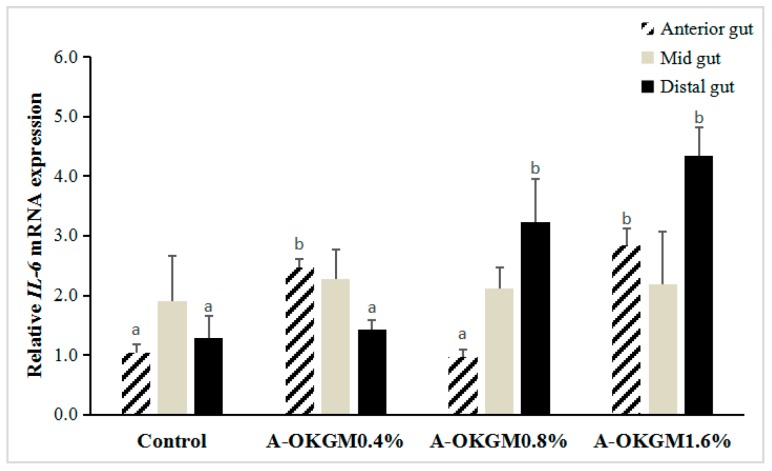
Effects of A-OKGM on *IL-6* expression in the anterior, mid- and distal gut of *Schizothorax prenanti*. The observed values are expressed as the mean ± S.E. ^a,b^ Means with different superscripts are significantly (*p* < 0.05) different from each other.

In the anterior intestine, *IL-6* expressions in the fish fed 0.4% and 1.6% A-OKGM diets was significantly higher (*p* < 0.05) than that in the control group (Figure 4). However, in the mid-intestine, there were no significant differences (*p* > 0.05) in *IL-6* mRNA levels among any of the groups following the feeding trials. In the distal intestine, the expressions of *IL-6* in fish fed the 0.8% and 1.6% A-OKGM diets was significantly higher (*p* < 0.05) than that in the control group.

## 3. Discussion

KGM consists of β-d-glucose and β-d-mannose monomers mainly connected by β-1,4-glycosidic bonds, with some side chains connected through β-1,3-glycosidic bonds [11]. KGM is a polysaccharide with prebiotic properties. It is well-established that host organisms exhibit better growth performance and higher expression of immunoregulatory genes after being administered prebiotics [12,13]. Recently, KGM has found application in diverse fields due to its structure, such as for film preparation in drug and food industries [14,15]. However, the high viscosity and molecular weight of KGM impede its application in the field of aquaculture. Hence it is necessary to produce a variety of KGM with lower viscosity and molecular weight. Our previous study showed that *S. prenanti* exhibited greater immunity and growth performance after being fed a diet supplemented with OKGM [16]. However, the viscosity and molecular weight of OKGM were still too high, and we further degraded OKGM with HCl to obtain A-OKGM. Onishi et al. [17] showed that pulverized dietary KGM suppressed allergic rhinitis-like symptoms in mice upon immunization and nasal sensitization with ovalbumin. Moreover, Chen et al. [18] reported that acid-hydrolyzed KGM exerted a greater prebiotic effect than non-modified KGM on BALB/c mice. In our present study, A-OKGM, as a potential prebiotic, had positive effects on the serum immune parameters and expressions of immunity-related genes in the gut of *S. prenanti.*

Both the innate and adaptive immune system have developed extensively in fish. Some specialized cells such macrophages, cytotoxic cells, and various proteins are critical for the innate immune system, as they protect fish from pathogens [19,20,21]. Lysozyme is plays an important role in the innate immune system; and occurs in both the vertebrates and invertebrates [22]. Staykov et al. [23] demonstrated that serum lysozyme activity in rainbow trout fed a 2 g·kg^−1^ mannon oligosaccharides (MOS) diet was significantly increased. The serum lysozyme activity of *Carassius auratus* gibelio was significantly higher in a treatment group fed 480 mg·kg^−1^ MOS than in the control group [24]. Moreover, *Labeo rohita* fed 100–500 mg·kg^−1^ β-glucan for 56 days also showed significantly higher serum lysozyme activity than control group fishes [25]. Dawood et al. [26] demonstrated that lysozyme activity was significantly higher in red sea bream fed a diet containing 0.1% β-glucan in combination with 0.025% HK-LP than in other groups. The molecular structure of A-OKGM is similar to those of MOS and β-glucan. In the present study, the serum lysozyme activity in fish fed the 1.6% A-OKGM diet was significantly increased compared to that in the control group.

CRP, which belongs to the pentraxin family of proteins, is commonly associated with the acute phase immune response [27,28], which is the first line of host defense against infection, injury, or trauma. This reaction aims at eliminating infective organisms and preventing further tissue damage, and also at restoring the host’s normal functions [29]. As CRP is an acute-phase protein, it functions in numerous immune-related activities, such as the killing of microbes, repair of tissue damage, destruction of potential pathogens, and restoration of the healthy state [30]. In many fish species, the concentration of CRP is quite high. However, its concentration will further increase by up to 20-fold after fishes are challenged with high temperature or inflammatory agents [31,32,33]. To date, inflammatory agents have been found to be mainly pathogens [34,35,36]. Studies on the effects of prebiotics, such as A-OKGM, on the level of CRP are limited. In carp, oral administration of β-glucan for 25 days significantly increased the serum CRP levels [37]. In the present study, the serum CRP levels in *S. prenanti* fed the A-OKGM diets were significantly higher than those in the control group, regardless of the dosage. The IgM content was also significantly increased, regardless of the dosage. Zhang et al. [16] demonstrated that the content of IgM in *S. prenanti* fed the 2000 mg/kg OKGM was significantly higher than that in other groups. IgM is the major serum immunoglobulin in teleost fish, and is an important molecule mediating the humoral immune responses [38]. As for the immune organs, the spleen and liver indexes were significantly increased after fish were fed 1.6% A-OKGM and 0.8% A-OKGM. Taken together, the present results indicated that diets supplemented with A-OKGM could to some degree enhance the immunity of *S. prenanti*.

To the best of our knowledge, our research is the first to explore the effects of A-OKGM on the expression of immune-related genes in the gut of *S. prenanti*. Our present findings indicate that the treatment diet stimulated the secretion of cytokines by immune cells in the fish gut. It is interesting to note that the expression of *TNFα* in the midgut and the distal gut increased steadily in a dose-dependent manner (0.4–1.6%). The *IL-6* mRNA level in the distal gut also increased in a dose-dependent manner. However, similar patterns were not observed for the expressions of *IL-1β*. 

*IL-1β*, which belongs to the interleukin-1 family, is secreted by various cells, such as macrophages, and T and B lymphocytes [39]. By promoting the growth of lymphocytes and stimulating the effector cells for immune and inflammatory responses, it can enhance cell mediated immunity [40]. In mammals, *IL-1β* mRNA expression in the ileal tissue of pigs fed with distillers dried grains with solubles (DDGS) was significantly higher than that in pigs fed with other diets. DDGS, which is obtained from ethanol plants, contains high levels of β-glucan and MOS [41]. In carp, diets supplemented with β-glucan can stimulate macrophages and promote *IL-1β* expression [42]. In the cod *Gadus morhua*, spleen cells incubated with β-glucan showed a significant up-regulation of *IL-1β* at 24 h post-incubation compared to control cells [43]. Guzmán-Villanueva et al. [44] demonstrated that *IL-1β* mRNA was significantly up-regulated in gilthead seabream fed β-glucan for four weeks, when compared with other groups. In the present study, 0.4% and 1.6% A-OKGM diets significantly enhanced *IL-1β* expressions in the anterior gut. *S. prenanti* fed 0.8% and 1.6% A-OKGM diets also showed significantly higher expression of *IL-1β* in the distal gut.

*IL-6* is a multifunctional cytokine with a wide range of biological activities associated with the regulation of immune responses and inflammation in many cell types, including macrophages and lymphocytes [45,46]. It is induced in response to viral or bacterial infections and pro-inflammatory cytokines, such as IL-1, tumor necrosis factor (TNF)-α, or platelet derived growth factor (PDGF) [47,48,49]. Similar to *IL-1β*, *IL-6* can act as a pro-inflammatory cytokine in innate immune responses. To stimulate inflammatory activity, *IL-6* promotes the production of acute phase response proteins, including the C-reactive proteins and the related serum amyloid A proteins [50]. Our present study indicates that A-OKGM up-regulates *IL-6*. Specifically, *IL-6* mRNA levels in the 0.4% and 1.6% A-OKGM diet groups were significantly increased in the anterior gut. The 0.8% and 1.6% A-OKGM diets significantly enhanced *IL-6* expression in the distal gut. In carp, a β-glucan enriched bath stimulated the wound healing process by significantly up-regulating *IL-6* expression after three days post-injury [51]. To date, most investigators have focused only on the changes in *IL-6* expression in response to bacterial challenge. Studies on the effects of prebiotics, such as A-OKGM, on *IL-6* expression are limited. Raida et al. [52] demonstrated that *IL-6* expression in rainbow trout was significantly induced following infection with *Yersinia ruckeri*. In *Oncorhynchus mykiss* larvae, *IL-6* was up-regulated after infection with *Ichthyophthirius multifiliis* and its expression slowly increased until the end of the experiment [53]. Zhang et al. [54] reported that the mRNA levels of *IL-6* in the spleen, intestinal tissues, and liver of blunt snout bream all increased significantly (*p* < 0.05), with maximum values attained at 6 h, 3 h, and 6 h (10-, 6-, and 18-fold increase, respectively) after the fishes were injected with *Aeromonas hydrophila*.

Because TNFα can eradicate various pathogens by activating the cellular response, *TNFα* is regarded as an important biomarker to indicate the health status of both mammals and fishes [55,56,57]. Many studies have reported that polysaccharides induce the expression of *TNFα* mRNA. In mammals, the *TNFα* expression in rats fed with *Grifola frondosa* polysaccharide was markedly increased [58]. In carp, Yuan et al. [59] reported that administration of *Astragalus* polysaccharides significantly up-regulated *TNFα* mRNA in carp. Similarly, Immunogen (mainly β-glucan and MOS) significantly enhanced *TNFα* gene expression in rainbow trout [60]. In the present study, in the anterior gut, the 0.4% and 1.6% A-OKGM diet groups showed significantly enhanced *TNFα* gene expression. Additionally, diets supplemented with 0.8% and 1.6% A-OKGM significantly promoted the expression of *TNFα* in the mid- and distal guts.

Thus, feeding an A-OKGM supplemented diet to *S. prenanti* induced an immune response in the gut. Although the mechanisms of action of A-OKGM on the gut immune system are unclear, we suspect that dietary A-OKGM is closely involved the production of immune cytokines. It has been clearly shown that A-OKGM promotes *IL-1β*, *IL-6*, and *TNFα* gene expression. Cytokines (such as *IL-1β*, *IL-6*, and *TNFα*) are important for the elimination of pathogens, and can induce essential cells, such as macrophages, and lymphocytes in the infected tissues [61]. Cytokines can also induce acute phase proteins, including mannose-binding lectin (MBL) and CRP [62]. An increased content of CRP in the serum was also observed in our study.

## 4. Materials and Methods

### 4.1. Preparation of A-OKGM

A-OKGM was produced through our previously described method [10].

### 4.2. Composition of Diets

The experimental diets are presented in Table 2. Different levels (0, 4.0, 8.0, and 16.0 g·kg^−1^) of A-OKGM were added into the diets to investigate its immune promoting properties, and the diets met all nutritional requirements for *S. prenanti*.

### 4.3. Feeding Trials

Two hundred fish were purchased from a local farm (Yuquantown, Tianquan, Yaan, Sichuan, China), and then transported to the Functional Food laboratory of Sichuan Agriculture University, where they were reared in 12 fiberglass tanks (50 cm × 70 cm × 40 cm). First, the 200 fishes were allowed to acclimatize to the new environment for three weeks. Then, 120 healthy fishes (initial weight 70.12 ± 1.56 g) were chosen for experimental use and were reared in 12 fiberglass tanks (10 fish per tank) at 20 ± 1 °C temperatures, under the natural photoperiod (12L:12D). Each diet was assigned to three tanks in a completely randomized manner. The diets were fed to the fish at a fixed ration (1% of the total fish weight per tank) three times daily. Fecal matter was quickly removed during the experiment. The feeding trial was performed for eight weeks. All procedures above were approved by Laboratory Animal Management Committee of Sichuan Province (permit number SYXK2015-196, 22 July 2015).

### 4.4. Sampling

After the feeding trial, all fishes were subject to starvation for 24 h to reduce the influence of diets on serum related parameters [7]. Blood samples were collected from the caudal veins of the fishes (three fish per tank). The blood samples were centrifuged at 3000× *g* for 10 min at 4 °C after clotting, and the supernatants were collected as serum, which was used for analysis of the IgM level, CRP level, and lysozyme activity. Four fishes from each tank were used for determination of the visceral index.

### 4.5. Biochemical Analysis

The visceral indexes were measured using the following equations:

- Spleen index = weight of spleen/body weight.
- Head kidney index = weight of head kidney/body weight.
- Gut index = weight of gut/body weight.
- Mesonephros index = weight of mesonephros/body weight.
- Liver index = weight of liver/body weight.

The fish CRP and IgM levels were determined using enzyme linked immunosorbent assay (ELISA) kits, purchased from Shanghai Yaji biotechnology Institute (Shanghai, China). Lysozyme activity was determined using a commercial kit (Nanjing, China) according to the manufacturer’s instructions. 

### 4.6. RT-qPCR

**Figure 5:**
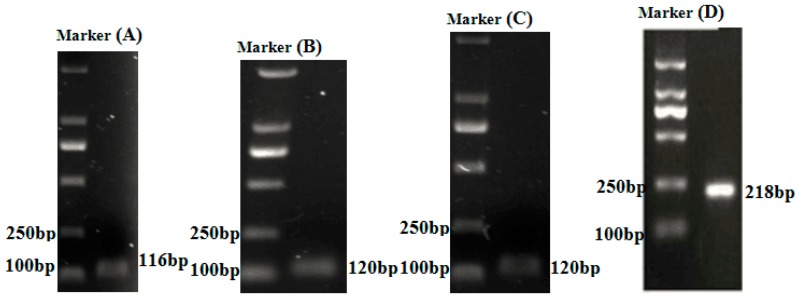
The amplification products of RT-PCR. Marker: DL2000, (**A**) *TNFα*, (**B**) *IL-1β*, (**C**) *IL-6*, (**D**) *β-actin*.

For RNA extraction, the anterior intestine, mid-intestine, and distal intestine of three fishes from each replicate tank were removed and pulverized with mortars under liquid nitrogen. Total RNA was extracted from the anterior, mid, and distal intestinal samples according to our previous method [10]. To analyze the expression of immune-related genes, quantitative real-time PCR (RT-PCR) was performed on the CFX96 system (Bio-Rad, Hercules, CA, USA) using SYBR Green (VazymE, Nanjing, Jiangsu, China). All of the primer sequences used in our present study are given in Table 3. RT-PCR was performed in a final volume of 10 μL, and each reaction mixture contained 5 μL of AceQ qPCR SYBR Green Master Mix (VazymE, Nanjing, Jiangsu, China), 0.25 μL of each primer, 1 μL of cDNA, and 3.5 μL of RNase-free H_2_O. The thermal program included 95 °C for 3 min, followed by 39 cycles of 95 °C for 10 s; and the annealing temperature of each gene for 30 s. After each run melting curves for each gene were constructed to confirm the specificity of the primers. All of the reactions were conducted in triplicate. The relative expression of the genes was calculated using the 2^−^^ΔΔ*C*t^ method, and *β-actin* was used as an internal control. Moreover, 2.0% agarose gel electrophoresis was used to ensure that each reaction contained the product of the correct size (Figure 5). The detail information of genes were shown in Tables S1−S3 and Figures S1–S3.

### 4.7. Statistical Analysis

Data are presented as the mean and standard error. One-way ANOVA and Duncan’s multiple range tests were chosen to analyze the data using SPSS 19.0 statistical software (SPSS Inc., Chicago, IL, USA). The minimum significance level was *p* < 0.05.

## 5. Conclusions

In conclusion, A-OKGM modified from KGM can act as an immunostimulant to enhance the immunity of *S. prenanti* by promoting serum immune parameters and the expressions of immune-related genes.

## Figures and Tables

**Table 1:** Effect of A-OKGM on visceral index of *S. prenanti*.

Group	Control	Acidolysis-Oxidized Konjac Glucomannan (A-OKGM)		
	1 (0)	2 (0.4%)	3 (0.8%)	4 (1.6%)
Head kidney index	0.0003 ± 0.00005	0.0004 ± 0.0001	0.0003 ± 0.00003	0.0003 ± 0.00002
Mesonephros index	0.0023 ± 0.00028	0.0026 ± 0.00031	0.0032 ± 0.00021	0.0027 ± 0.00044
Spleen index	0.0014 ± 0.00013 ^a^	0.0011 ± 0.00024 ^a^	0.0011 ± 0.00024 ^a^	0.0023 ± 0.00036 ^b^
Gut index	0.0122 ± 0.00112	0.0118 ± 0.00056	0.0127 ± 0.00111	0.0139 ± 0.00033
Liver index	0.0140 ± 0.0007 ^a^	0.0147 ± 0.00005 ^a^	0.0166 ± 0.00031 ^b^	0.0151 ± 0.00023 ^a^

The observed values are expressed as the mean ± S.E. ^a,b^ Means within different superscripts are significantly (*p* < 0.05) different from each other.

**Table 2:** Formulation of experimental diets.

Diets Component	Control	A-OKGM		
Formulation (%)	A 0%	A 0.4%	A 0.8%	A 1.6%
A-OKGM	0	0.40	0.80	1.60
Fish meal	42.00	42.00	42.00	42.00
Rapeseed oil	3.00	3.00	3.00	3.00
Soybean meal	21.00	21.00	21.00	21.00
Flour	20.00	20.00	20.00	20.00
Starch	10.00	9.60	9.20	8.40
Bran	1.00	1.00	1.00	1.00
Vitamin premix + choline ^a^	0.50	0.50	0.50	0.50
Mineral premix ^b^	1.00	1.00	1.00	1.00
Ca(H_2_PO_4_)_2_	1.50	1.50	1.50	1.50
Total	100	100	100	100
Nutrition level (%)				
Crude protein	35.2	35.2	35.2	35.2
Crude lipid	8.19	8.19	8.19	8.19
TE (MJ/kg) ^c^	16.53	16.46	16.39	16.31
Ca	2.02	2.02	2.02	2.02
P	1.52	1.52	1.52	1.52
Lys	2.87	2.87	2.87	2.87
Met + Cys	1.32	1.32	1.32	1.32

^a^ Vitamins provided per kg of diet: VA 5000 IU; VD 1000 IU; VE 30 IU; VK 2.5 mg; VB_1_ 5 mg; VB_2_ 8 mg; VB_6_ 7 mg; VB_12_ 0.01 mg; niacin 30 mg; pantothenic acid 25 mg; folic acid 0.5 mg; biotin 0.2 mg; VC 35 mg; Inositol 50 mg; choline chloride 700 mg. ^b^ Minerals provided per kg of diet: Mn 10 mg; Zn 30 mg; Fe 60 mg; Cu 3 mg; I 1 mg; Se 0.2 mg. ^c^ Total energy (TE) is the calculated value. Other nutrient levels are measured values.

**Table 3:** Primers used in real-time polymerase chain reaction (RT-PCR) analysis.

Genes	Forward Primer (5′–3′)	Reverse Primer (5′–3′)	Product Size (bp)	Annealing Temperature
*TNFα*	TGTCTGCTTCACGCTCAACA	AATGGATGGCWGCCTTGGA	116	59 °C
*IL-1β*	GGTGGTGAACATCATCATTGC	AGACGCTCTTCGATCACATTC	120	55.7 °C
*IL-6*	CCACCTGTAACCATAAGAAAAGAAC	TTGCTCAAAATCTGTCCCCAT	120	59.5 °C
*β-actin*	GATTCGCTGGAGATGATGCT	CGTTGTAGAAGGTGTGATGCC	218	55.8 °C

## Supplementary Materials

Supplementary materials can be found at www.mdpi.com/1422-0067/18/12/2558/s1.
