# Supplementary material for: Effect of Dietary Acidolysis-Oxidized Konjac Glucomannan Supplementation on Serum Immune Parameters and Intestinal Immune-Related Gene Expression of Schizothorax prenanti
Source: Int J Mol Sci. 2017 Nov 28;18(12):2558. doi: 10.3390/ijms18122558 (PMC5751161; doi:10.3390/ijms18122558)
Supplement: Supplementary file 1 [file ijms-18-02558-s001.pdf]

```
1    GGTGGTGAACATCATCATTGCTGTGGAAAGATTGAAGCACATCAAAGTGATGTCTTCTGA
1     V  V  N  I  I  I  A  V  E  R  L  K  H  I  K  V  M  S  S  D
61   CAAGATTTGTGAAGCTGCGCTGCTCAACTTCTTCTTGGAGAATGTGATCGAAGAGCGTCT
21    K  I  C  E  A  A  L  L  N  F  F  L  E  N  V  I  E  E  R
```

Figure S1. The partial nucleotide sequence of *IL-1β* gene and the deduced amino acids sequence of *Schizothorax prenanti*.

```
1    TGTCTGCTTCACGCTCAACAAGTCTCAGAACAATCAGGAAGGTGGAAATGAGCTCAGGCT
1     V  C  F  T  L  N  K  S  Q  N  N  Q  E  G  G  N  E  L  R  L
61   CACATTAAGAGATCATCTTTCAAAAGAAAATGCCACTTCCAAGGCTGCCATCCATT
21    T  L  R  D  H  L  S  K  E  N  A  T  S  K  A  A  I  H
```

Figure S2. The partial nucleotide sequence of *TNFα* gene and the deduced amino acids sequence of *Schizothorax prenanti*.

```
1    AACTTTCAGACGTGTTTTGCGTGCTGTCACTCAAACTACTGGACAGGAGGAAGAGGTCTT
1     N  F  Q  T  C  F  A  C  C  H  S  N  Y  W  T  G  G  R  G  L

61   TGGTGTCACCATGGGGACAGATTTTGAGCAAA
21    W  C  H  H  G  D  R  F  *  A
```

Figure S3. The partial nucleotide sequence of *IL-6* gene and the deduced amino acids sequence of *Schizothorax prenanti*.

Table S1. The similarity of predicted amino acid sequences of *Schizothorax prenanti* *IL-1β* to other fish.

Species	Amino Acid Sequence Similarity	GenBank Accession No.
Carassius auratus	87%	AGU42184.1
Cyprinus carpio	79%	CAB52366.1
Ctenopharyngod idella	56%	AER38414.2
Danio rerio	46%	CAR66436.1
Ictalurus punctatus	45%	CAE51868.1
Dicentrarchus labrax	44%	CAC41006.1
Chionodraco hamatus	38%	CAD92853.1

Table S2. The similarity of predicted amino acid sequences of *Schizothorax prenanti* *TNFα* to other fish.

Species	Amino Acid Sequence Similarity	GenBank Accession No.
Percocypris pingi	94%	AIN25992.1
Carassius auratus	92%	AIL81467.1
Cyprinus carpio	92%	AFQ20281.1
Cirrhinus mrigala	84%	AGE13928.1
Pimephales promela	76%	AFG17065.1
Ctenopharyngod idella	74%	ABU88424.1
Danio rerio	68%	AAR06286.1
Megalobrama amblycephala	66%	AII20593.1

Table S3. The similarity of predicted amino acid sequences of *Schizothorax prenanti* *IL-6* to other fish.

Species	Amino Acid Sequence Similarity	GenBank Accession No.
Sinocyclocheilus rhinocerous	97%	XM016520587.1
Sinocyclocheilus grahami	92%	XM016267780.1
Cyprinus carpio	89%	LN590989.1
Danio rerio	79%	NM001330258.1
